# Polycomblike protein PHF1b: a transcriptional sensor for GABA receptor activity

**DOI:** 10.1186/2050-6511-14-37

**Published:** 2013-07-23

**Authors:** Shamol Saha, Yinghui Hu, Stella C Martin, Sabita Bandyopadhyay, Shelley J Russek, David H Farb

**Affiliations:** 1 Department of Pharmacology & Experimental Therapeutics, Laboratory of Molecular Neurobiology, Boston University School of Medicine, Boston, MA 02118, USA; 2 Department of Pharmacology & Experimental Therapeutics, Laboratory of Translational Epilepsy, Boston University School of Medicine, 72 East Concord Street, Boston, MA 02118, USA

## Abstract

**Background:**

The γ-aminobutyric acid (GABA) type A receptor (GABA_A_R) contains the recognition sites for a variety of agents used in the treatment of brain disorders, including anxiety and epilepsy. A better understanding of how receptor expression is regulated in individual neurons may provide novel opportunities for therapeutic intervention. Towards this goal we have studied transcription of a GABA_A_R subunit gene (*GABRB1*) whose activity is autologously regulated by GABA via a 10 base pair initiator-like element (β_1_-INR).

**Methods:**

By screening a human cDNA brain library with a yeast one-hybrid assay, the Polycomblike (PCL) gene product PHD finger protein transcript b (PHF1b) was identified as a β_1_-INR associated protein. Promoter/reporter assays in primary rat cortical cells demonstrate that PHF1b is an activator at *GABRB1,* and chromatin immunoprecipitation assays reveal that presence of PHF1 at endogenous *Gabrb1* is regulated by GABA_A_R activation.

**Results:**

PCL is a member of the Polycomb group required for correct spatial expression of homeotic genes in *Drosophila*. We now show that PHF1b recognition of β_1_-INR is dependent on a plant homeodomain, an adjacent helix-loop-helix, and short glycine rich motif. In neurons, it co-immunoprecipitates with SUZ12, a key component of the Polycomb Repressive Complex 2 (PRC2) that regulates a number of important cellular processes, including gene silencing via histone H3 lysine 27 trimethylation (H3K27me3).

**Conclusions:**

The observation that chronic exposure to GABA reduces PHF1 binding and H3K27 monomethylation, which is associated with transcriptional activation, strongly suggests that PHF1b may be a molecular transducer of GABA_A_R function and thus GABA-mediated neurotransmission in the central nervous system.

## Background

The γ-aminobutyric acid (GABA) type A receptor (GABA_A_R) plays a critical role in the pathophysiology of brain disorders such as anxiety and epilepsy, presenting an important therapeutic target for research. Of particular interest is the mechanism that underlies the expression of eight distinct GABA_A_R subunit classes whose collection of genes are differentially transcribed to form diverse receptor subtypes, with variable affinities for activation and modulation [1]. Variations in receptor subunit composition are also associated with different disease states. For instance, pilocarpine induces status epilepticus (SE) and spontaneous seizures in rats that are accompanied by a decrease in α_1_ and β_1_ subunit mRNAs, and a marked increase in α4 [2]. Recent reports using chromatin immunoprecipitation (ChIP) assays of primary neurons and slices of dentate gyrus from animals 24 hours after SE have shown that levels of these GABA_A_ receptor subunits may be regulated by changes in transcription that are driven by activity-dependent transcription factors [3-6].

Most interesting to the study of GABA_A_R regulation is the fact that chronic activation leads to an associated decrease in the levels of particular GABA_A_R subunit mRNAs, their cognate proteins, and their promoter/reporter activity, as measured in primary cultured neurons and *in vivo*. While it is certainly well established that the majority of genes rely on upstream regulatory elements to control relevant levels of gene expression, our previous studies showed that an initiator (β_1_initiator element [INR]; a 10 base pair (bp) core sequence that contains the transcriptional start site of *GABRB1*) is critical for the expression of β_1_ subunit mRNAs in neocortical and hippocampal neurons [7]. In fact, sequential deletion of most of the *GABRB1* promoter (*GABRB1-p*) reveals that the initiator is indispensable for neuron-specific promoter activity that is autologously regulated (transcriptionally repressed after chronic GABA_A_R activation). Replacing the core *GABRB1-p* with three concatenated copies of the β_1_-INR reconstitutes full promoter/reporter activity that is both neural-specific and autologously regulated.

Transcriptional regulators function through DNA-protein or protein-protein interactions that regulate the recruitment and assembly of the pre-initiation complex (PIC), which contains RNA polymerase II and general transcription factors (GTFs), TFIID, B, A, E, F and H [8-11]. The TATA box is located nearly −30 nucleotides upstream of the transcription start site and directs the initiation of transcription and assembly of the general transcription apparatus [12]. The downstream INR contributes to start site selection and directs the transcriptional initiation of genes with non-canonical TATA boxes [13]. Although a number of INRs have been identified among mammalian genes, the initiator binding complex is poorly understood. RNA polymerase II recognizes core promoter sequences to influence start site selection at the core promoter [14,15]. TAF_II_250 and TAF_II_150 (TAFs 1 and 2) [16] contribute to the selective recognition of promoters containing INRs [17-21]. A number of transcriptional regulators such as TFII-I, E2F, YY1 and USF stimulate transcription by binding to sites that overlap core promoter sequences [12,22]. Specific INR-binding proteins like YY1 and TFII-I contain distinct motifs for DNA binding [23-25]. YY1 binds through two zinc finger (C2H2) domains [26] whereas TFII-I, a context-dependent DNA recognition protein, binds through multiple helix-loop-helix (HLH) motifs with the aid of a basic rich region [24].

Chromatin remodeling plays an important role in either facilitating or preventing RNA polymerase II access to promoter regions, targeting N-terminal histone tails for acetylation, methylation, phosphorylation and/or ubiqutination modification(s) [27-30]. Two groups of proteins are found to be involved in regulating the modification status of chromatin at promoter regions: Trithorax (trxG) and Polycomb-Group (PcG) proteins. Both protein groups maintain active and silent status of transcriptional activity, respectively [31,32]. PcG proteins are encoded by some 40 genes in *Drosophila*, which include Polycomb (PC), Polyhomeotic (PHO), Polycomblike (PCL) and Posterior sex comb (PSC). PcG proteins maintain promoters in an inactive state, whereas trxG proteins counteract silencing by stimulating transcription [28,32-34]. Recently, two main Polycomb groups of repressive complexes have been characterized: PRC1 and PRC2, which appear to form biochemically distinct repressive units. Four core components of PRC2 are EZH2, SUZ12, EED and RbAp46/48 [32] and each protein in the complex has a distinct functional role in silencing transcriptional activity.

In this paper, we now show that PHF1b, a Polycomblike protein, binds to the β_1_-INR to stimulate transcription. In addition, our results demonstrate that chronic GABA treatment reduces the presence of PHF and monomethylated histone H3 lysine 27 (H3K27) at endogenous rat *Gabrb1-p*, consistent with a role for PHF1b in remodeling the local chromatin environment of the core promoter region in response to neuronal signals.

## Results

### Isolation of a cDNA encoding an initiator-associated protein

**Figure 1:**
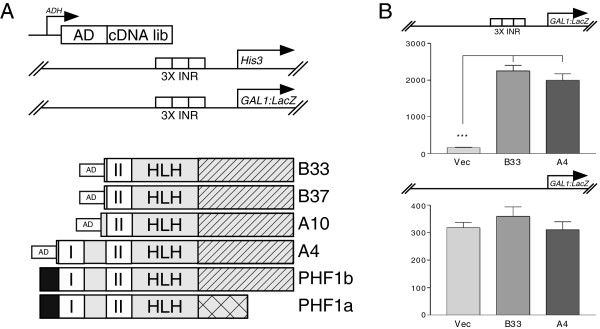
**Results of a yeast one-hybrid assay using expressed human brain cDNAs and β**_**1**_**-INR sequences. A)** The NLY2 yeast strain was transformed to contain two integrated reporters (*His*3 and *Lac*Z) (top panel) under the control of three tandem β_1_-INRs. This strain was used to screen a yeast expression library containing cDNAs derived from human adult or fetal brains. cDNAs were expressed from a yeast 2 micron based multi-copy plasmid with expression controlled by the *ADH* promoter. The libraries of expressed proteins contain a GAL4 activation domain (AD) fused to each cDNA in frame to facilitate one-hybrid screening in yeast. Transformed yeast colonies were screened for their ability to grow on selective solid media containing 10 mM 3-aminotriazole and lacking histidine. To confirm clone selection, expression of β-galactosidase was measured by plating yeast colonies on plates containing chromogenic dye (X-gal). Purified clones are shown in relation to the wild type and full length PHF1b (human Polycomblike protein) and PHF1a sequence. Two plant homeodomain (PHD) fingers are shown (I or II) with white boxes. Black box represents the amino terminus. Different carboxyl termini that result from alternative splicing are shown with hatched (PHF1b) and cross-hatched bars (PHF1a). A putative helix-loop-helix forming sequence is depicted by “HLH”. **B)** Screened candidates require INR sequence for reporter gene activation. Top panel shows candidates A4 and B33 activates *β-galactosidase* reporter gene only when reporter promoter contains the INR sequence. A reporter without INR sequence (bottom panel) shows no activity from the candidates in comparison to the vector plasmid. Results are expressed as mean values ± SEM.

To further understand neural-specific expression of *GABRB1-p*[7], we cloned the factor that associates with the β_1_-INR. A one-hybrid screen was performed using a transformed *S. cerevisiae* strain (Figure 1A, top panel) that included two chromosomally integrated reporters and a human neonatal or adult brain cDNA library. Each reporter gene (*His*3 and *Lac*Z) was regulated by three tandem repeats of the β_1_-INR, a configuration shown to reconstitute neuronal specificity and autologous regulation of *GABRB1*[7]. In a parallel experiment, nonselective media containing no aminotriazole was used to make sure that enough yeast transformants were obtained to cover the complexity of the cDNA library.

Five hundred yeast colonies carrying potential candidates were isolated from the *His*3 screen. These yeast colonies were further tested for their ability to express the second reporter *Lac*Z, which yielded 50 candidates that stimulated both reporters. Only four candidates (A4, A10, B33, B37) were positive upon retransformation of isolated clones. DNA sequencing and blast analysis revealed a perfect match of these four clones to a splice variant (b) of human Polycomblike protein PHF1. The full length PHF1b sequence in the database (ACC# BC008834) was compared to that of the isolated clones (see Figure 1, bottom panel). Candidates B33 and B37 are identical, whereas, candidate A10 is 10 amino acids longer than either B33 or B37. Candidate A4 lacks amino terminus of the PHF1b but contains two plant homeodomains (PHD) with the remainder of the carboxyl terminus. From the sizes of the clones and their amino terminus sequences, it was predicted that the zinc finger domain II with adjacent sequences is required for DNA recognition. This zinc finger is characterized by a C_4_HC_3_ motif and is found predominantly in proteins that are associated with chromatin remodeling [35]. The 120 amino acid carboxyl terminal region adjacent to the PHD finger II is predicted to form an HLH structure [36] whereas the carboxyl terminus showed no significant homology to any known structures. PHF1a, an alternatively spliced version of PHF1 [37], shares an identical amino terminus region comprising both finger domains and the HLH, but differs in the carboxyl terminus due to a frame shift that gives rise to two unique ends of different sizes (Figure 1A, bottom panel). Surprisingly, none of the candidates isolated were PHF1a. A confirmatory experiment for INR site dependency demonstrated that candidates A4 and B33 require INR sequences for *β-galactosidase* reporter gene activity (Figure 1B, top panel). Absence of the INR sequence in this promoter/reporter construct does not support activity (Figure 1B, bottom panel).

### PHD II and HLH are necessary for recognition of the β_1_-INR

PHD fingers are protein domains consisting of two zinc ions coordinated by cysteine and histidine residues in a C4HC3 motif [38,39]. Thus far, no specific function for this motif has been identified, however, it has been proposed that proteins containing PHD fingers are involved in processes of chromatin remodeling [35].

**Figure 2:**
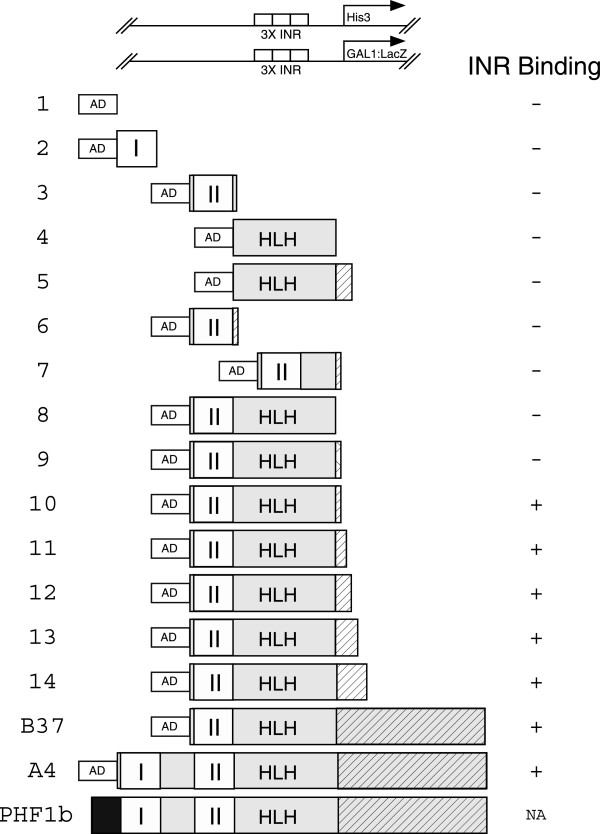
**Mapping sequences that are required for PHF1b DNA recognition.** Depicted is the yeast strain with chromosomally integrated reporters carrying three tandem β_1_ initiator sites that was used for one-hybrid assays. Candidates identified by one-hybrid assays were used to construct a number of 5′ and 3′ sequential deletions of the PHF1b gene to define the minimal β_1_-INR binding domain. The GAL4 AD was fused to all of the PHF1b fragments and tested for its ability to activate three tandem β_1_-INR sites linked to either *His*3 or *Lac*Z. Full length PHF1b is shown for comparison to relate relative sizes of AD fused PHF1b fragments (#2-14 with the exception of #9, which is a derivative of PHF1a) and one-hybrid candidates B37 and A4. The DNA binding activities both positive (+) and negative (−) are as indicated. The criterion for DNA binding is measured by the ability of the constructs to activate both reporter genes (*His*3 and *Lac*Z ) at levels comparable to B37 and A4 isolates. *His*3 expression was measured by the survival of the yeast colonies that could grow on plates that contained 10 mM 3-aminotriazole and β-galactosidase activity from the second reporter, measured by β-galactosidase assays. The results from both assays were the same and are indicated by one column to the right.

To determine the minimum sequence of PHF1b necessary for INR recognition, a series of PHF1b truncation mutants were engineered (Figure 2). The GAL4 activation domain (AD) [40] was fused to zinc finger domains I, II, and to the predicted HLH part of the protein [36]. The DNA binding activity of these cDNA products was tested in yeast. A number of cDNAs containing sequential deletions from the C-terminus were generated. Expression of cDNAs encoding either PHD I, II or HLH domains were not sufficient for DNA association as measured by growth on 10 mM 3-aminotriazole containing media and ability to activate the β-galactosidase reporter gene (Figure 2, rows 2, 3 and 4). A PHF1b fragment that terminated at the divergent sequence with PHF1a (Figure 2, row 8) [37] was also inactive. However, a longer version of PHF1b (Figure 2, row 14) that included an additional 40 amino acids beyond the putative HLH sequence was sufficient for growth support mediated by the β_1_-INR. Further sequential deletions defined the PHD finger II, HLH, and a portion of the 40 amino acid domain (11 amino acid region) as being the required sequence for INR recognition (Figure 2, row 10). The importance of the 11 amino acids (SFPSGQGPGGG) (glycine-rich motif) was further tested in the context of either the PHD finger II or HLH domain. The 11 amino acid sequence was fused to the end of PHD finger II (Figure 2, row 6) and to the HLH domain where the 11 amino acid sequence was imbedded in a larger sequence (Figure 2, row 5). Both fusion proteins (rows 5 and 6) failed to support β_1_-INR recognition as measured by the yeast one-hybrid assay. Similarly, the HLH alone was ineffective for INR recognition (Figure 2, row 4).

It is apparent from the one-hybrid assays that finger II, HLH and the 11 amino acids beyond the frame shift point of PHF1 proteins are essential for DNA recognition. The significance of the glycine-rich 11 amino acid motif in the context of the HLH domain is not clear. The alternatively spliced version PHF1a does not contain the glycine-rich sequence and does not bind β_1_-INR (Figure 2, row 9). The minimum PHF1b DNA binder requires 11 amino acids beyond the frame shift point of PHF1a (Figure 2, row 10). It is plausible that the 11 amino acids of PHF1b may not be essential for DNA binding but may be required for some structural stability of the protein.

### PHF1 proteins are localized to the nucleus

**Figure 3:**
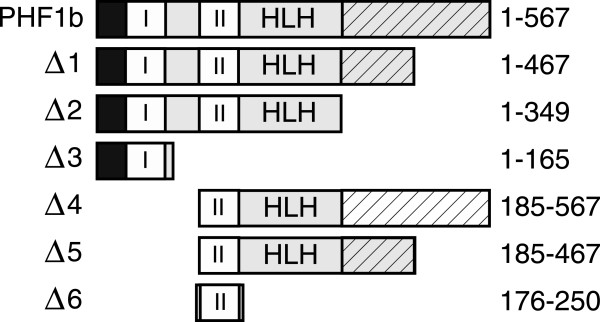
**PHF1b deletion derivatives.** PHF1b derivatives showing amino terminus and carboxyl terminus deletions. These deletions are used as GFP and GAL4(1–100) fusions to determine the nuclear localization and repressor domains of the protein. Numbers represent the amino acid positions that specify the sizes of the proteins.

**Figure 4:**
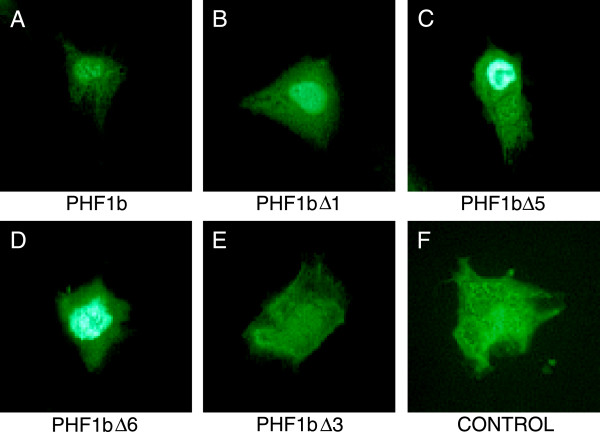
**PHD domain II is necessary for the localization of PHF1b to COS cell nuclei.** COS cells were transfected with GFP-PHF1b fusion constructs (see Figure 3 for PHF1b deletion derivatives). Control represents a vector expressing only GFP protein.

The larger *Drosophila* homolog of PHF1/PCL1 proteins is localized in the nucleus [41], but the nuclear localization motif has not yet been identified. To determine the region of PHF1b that contains the nuclear localization signal, a number of truncated versions of PHF1b were constructed (Figure 3) and fused to the green fluorescent protein (GFP) protein. Complementary DNAs coding for GFP-PHF1b fusion proteins were transfected into COS-7 cells that were fixed with paraformaldehyde. The transfected cells were viewed through a blue filter to detect the green fluorescence from the hybrid proteins. PHF1b Δ6 (Figure 4D) is sufficient for nuclear localization when compared with full length PHF1b (Figure 4A) or other PHF1b derivatives that contained amino terminus, PHD finger I and part of the carboxyl terminus of the protein (PHF1b Δ1 and Δ5) (Figure 4B and C). Taken together with the fact that the GFP fusion protein with the amino terminus and PHD Finger I (PHF1b Δ3) (Figure 4E) did not localize to the nucleus, as compared with GFP alone (Figure 4F), the nuclear localization sequence (NLS) is most likely located within the PHD finger II region and facilitates an association with DNA by localizing the protein in the nucleus.

**Figure 5:**
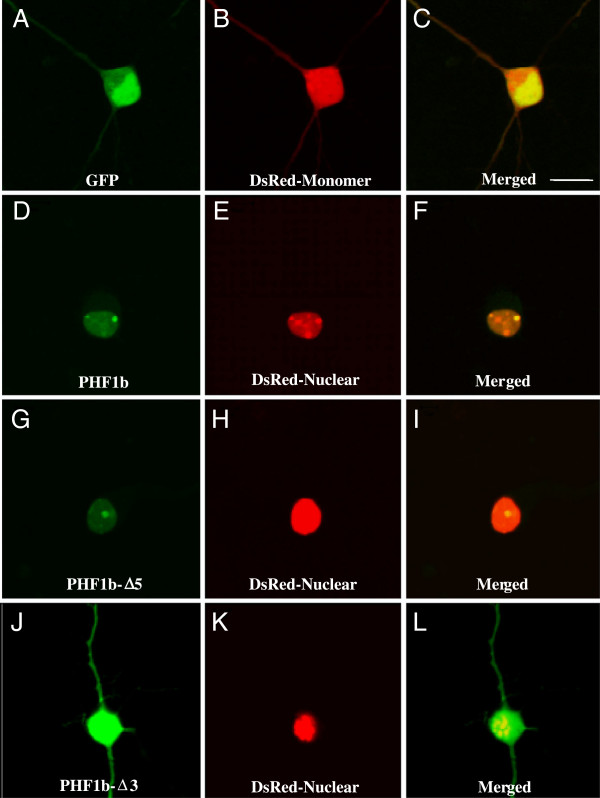
**PHF1b protein nuclear localization in rat neocortical neurons.** Primary rat neocortical neurons isolated from E18 brain and maintained one week *in vitro* were transfected with GFP-PHF1b fusion plasmids (CMV-GFP-PHF1b, CMV-GFP-PHF1b-Δ5, CMV-GFP-PHF1b-Δ3, see Figure 3) and examined 48 hours after transfection by confocal microscopy for nuclear localization relative to DsRed-Nuclear marker (CMV-DsRed-Nuclear), a red fluorescent protein that localizes to the nucleus. Control transfection of CMV-GFP **(A, C)** and CMV-DsRed-Monomer **(B, C)** construct expression is throughout cortical cells and expression is not restricted to the nucleus **(C)**. Both GFP-PHF1b **(D, F)** and GFP-PHF1b-Δ5 **(G, I)** fusion construct expression coincides with DsRed-Nuclear **(E, F and H, I)** indicating that both the PHF1b and the PHF1b-Δ5 protein contain a nuclear localization signal. In contrast, the GFP-PHF1b-Δ3 **(J, L)** fusion construct expression is not restricted to the nucleus **(DsRed-Nuclear, K, L)** suggesting that the nuclear localization signal of PHF1b is not localized at the N-terminus of the PHF1b protein. Scale bar is 10 μm.

In addition to the COS-7 nuclear localization study, we also investigated the localization of the same GFP-PHF1b fusion proteins in primary rat neocortical neurons (Figure 5). Results of confocal microscopy using transfected neurons shows a similar pattern of PHF1b expression as was observed with COS-7 cells. Full length PHF1b (Figure 5D, PHF1b) is restricted to the nucleus. Location of the nucleus was visualized by co-transfection of cytomegalovirus (CMV)-DsRed-Nuclear (Figure 5E, H and K). The PHD finger II domain is sufficient for nuclear localization (Figure 5G, PHF1b Δ5). GFP alone (Figure 5A), PHF1b Δ3 (Figure 5J) and DsRed-Monomer (Figure 5B) are not restricted to the nucleus. As compared to COS-7, the GFP-PHF1b expression pattern is highly restricted within the larger neuronal nucleus (Figure 5D).

### PHF1b stimulates *GABRB1* promoter activity in transfected primary cultured neurons

**Figure 6:**
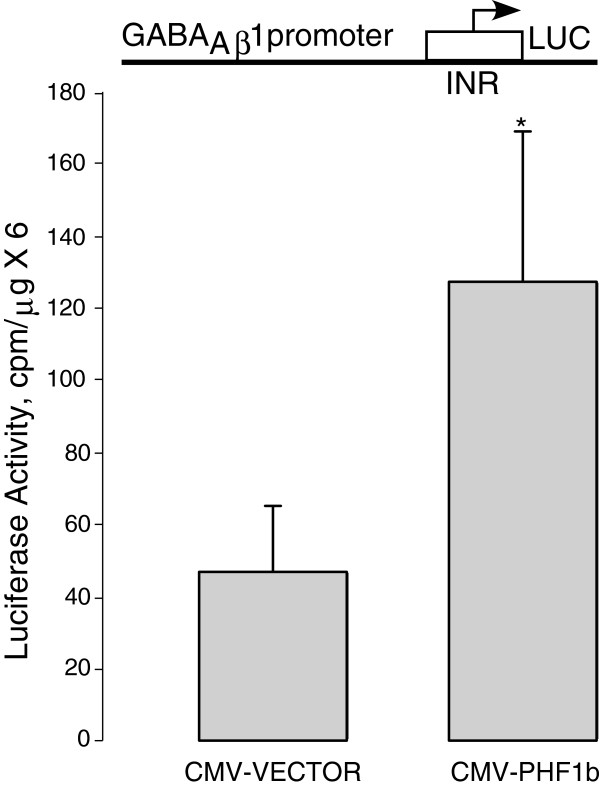
**Transient transfection assays using primary rat neocortical neurons.** The human β_1_ promoter (475 bp) fused to a luciferase reporter was co-transfected with an expression construct for either full-length PHF1b or an empty vector. The CMV promoter was used for PHF1b over-expression. (*) indicates significance of (p < .05), Student’s T test. Results are presented as mean values ± SEM (n=7). Luciferase counts were normalized to mg protein/dish.

DNA alignment of the human, mouse, and rat β_1_ promoters shows that the initiator sequence is identical and the region 192-bp upstream and downstream of the initiator is 94% similar (data not shown), suggesting that the key regulatory factors for the promoter are conserved across species. Considering the conserved nature of β_1_ promoters, the study of a human *GABRB1* promoter in rat primary neuronal cultures is quite likely to be relevant to gene regulation in humans. Towards this goal, an expression construct containing the human PHF1b cDNA under control of the CMV promoter was co-transfected into primary rat neocortical neurons to monitor the effects of such expression on human *GABRB1-p/*luciferase reporter activity. There is a 3-fold stimulation of *GABRB1* promoter activity observed upon PHF1b overexpression (Figure 6), suggesting that PHF1b may be an important regulatory factor of human *GABRB1* transcription.

### Chronic GABA exposure regulates PHF1 binding to endogenous *Gabrb1*

**Figure 7:**
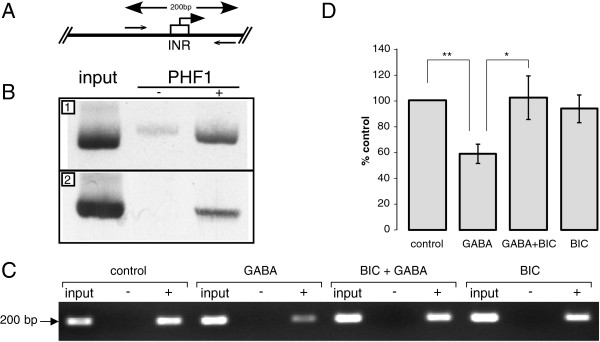
**Association of PHF1 proteins with endogenous *Gabrb1 *in neurons.** ChIP assays were performed using a PHF1(a and b) specific antibody and precipitated genomic DNA was found to contain the core promoter region of *Gabrb1* in primary rat neocortical neurons. Detection of the endogenous *Gabrb1* promoter was accomplished by PCR as depicted in **(A)** using two primers (arrows) that flank the β_1_-INR. The size of the PCR fragment is indicated above. Initiator position is depicted with a box and arrow showing the direction of transcription. **(B)** Bottom panel shows the presence or absence of *Gabrb1*-specific PCR products in fragments of genomic DNA that have been precipitated after addition of PHF1 antibodies. ChIP substrates are as indicated (1) primary neocortical neurons and (2) primary hippocampal neurons cultured for 7 days from E18 rat brains. **(C)** Representative data showing the presence or absence of *Gabrb1*-specific PCR products from ChIP performed with PHF1 antibody. Primary neocortical neurons were treated with either GABA (500 μM), GABA and the specific GABA_A_R antagonist bicuculline (50 μM), bicuculline alone, or relevant vehicle for 48 h, as described in Russek et al [7]. Presence of IgG in reaction is represented as “-“ and PHF1 antibody as “+”. **(D)** Quantitation of ChIP data displayed in **(C)** is represented as mean ± SEM and expressed as percent increase from control (% control). (*=significantly different from control, p < 0.05). All samples were analyzed as ratios of PHF1 antibody/IgG after normalization to input.

ChIP was used to demonstrate that PHF1 proteins are bound to the endogenous rat *Gabrb1* core promoter of both hippocampal and neocortical neurons (Figure 7B) where high levels of endogenous β_1_ subunit mRNAs have been reported [7]. Binding of PHF1 to *Gabrb1* is decreased after chronic treatment with GABA at a concentration reported to down-regulate β_1_ mRNAs and subunit levels in cultured neocortical neurons (Figure 7C and D). Moreover, blockade of GABA_A_Rs by the antagonist bicuculline reverses GABA-induced removal of PHF1 from *Gabrb1* suggesting that the effects of GABA exposure are through the GABA_A_ receptor*.*

**Figure 8:**
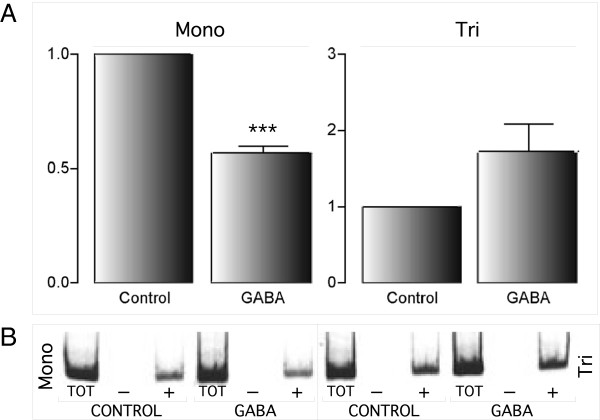
**GABA decreases H3K27 mono methylation on the *Gabrb1 *promoter.** Real time PCR analysis of ChIP assays show a statistically significant (***) decrease (n=5) of H3K27 monomethylation at the core promoter region (P = 0.0001) **(A)** with no significant change in the status of trimethylation (n=5, p=0.1184). Y-axis represents relative signal of H3K27 (mono- or tri-) methylation as compared to vehicle control (set as 1). A Student’s t-test was used to investigate the statistical significance of mono- and trimethylation. **(B)** Radiographic display showing amplified promoter fragments immunoprecipitated with H3K27me1 (mono) and H3K27me3 (tri) antibodies. Total input DNA and IgG lanes are marked by “TOT” and “-“, respectively. Results are expressed as mean values ± SEM.

A PRC2 complex protein EZH2 requires PHF1a (Pcl1) for efficient catalysis of (H3K27) trimethylation [42]. To determine if PHF1b functions in this regard at *Gabrb1*, we examined whether the trimethylation status of H3K27 is altered at the core promoter region after GABA treatment. No significant change in trimethylation at the H3K27 position was detected, however, there was a 26% decrease of monomethylation (n=6, p=0.0011) (Figure 8).

### TAF1 and TAF2 as co-activators of PHF1b at the *GABRB1* promoter

**Figure 9:**
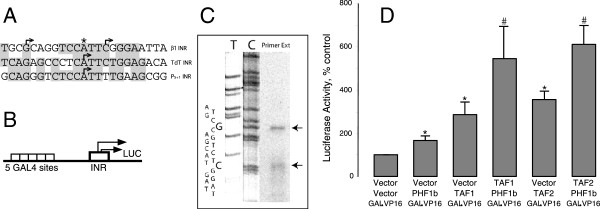
**Study of the β**_**1**_**-INR in COS cells. A)** Sequence similarities between β_1_-INR, TdT INR and the adeno-associated virus (AAV) P5+1 INR. Shadowed boxes highlight sequence identity. Arrows indicate transcription start sites. Star symbol indicates the major start site of β_1_-INR in neocortical neurons (7). **B)** Depiction of a TATA-less synthetic promoter/reporter construct carrying a single β_1_-INR with a GAL4 UAS (p5XG-β_1_-INR-Luc). Promoter activity of the construct is regulated by co-expression of an upstream activator (GAL4-VP16). Arrows show the direction of transcription. **C)** Primer extension analysis of RNA from COS-7 cells transfected with the p5XG-β_1_-INR-Luc construct. Primer extension products are separated on a sequencing gel. Sequencing reactions (dC and dT) were run alongside of primer extension products to determine the exact start sites for initiation. Top strand DNA sequence of β_1_-INR is also shown next to sequencing lanes. Arrows indicate the major initiation sites in COS-7 cells. **D)** Co-activation of PHF1b transcriptional activity by TAF1 and 2. COS-7 cells were co-transfected with p5XG-β_1_-INR-Luc, GAL4-VP16 and combinations of PHF1b, TAF1 and TAF2. 48 hours after transfection, cells were harvested and assayed for luciferase activity. Results shown are mean values ± SEM and normalized to protein content within each dish as well as to vector control (Vector+Vector+GAL-VP16 defined as 100%). “*” indicates significantly different from vector control (p < 0.05) as determined by 95% confidence interval. “#” indicates significantly different from PHF1b (Vector+PHF1b+GAL-VP16) (p < 0.05) as determined by 95% confidence interval.

TAF1 and TAF2 contribute to DNA binding and core promoter selectivity of RNA pol II [17]. It has been shown that the complex formed by TAF1 and TAF2 preferentially bind to INR-like DNA sequences compared to random DNA [21]. Independently, these two TAFs do not show DNA sequence specificity, but as a complex they recognize DNA and thereby recruit TFIID to TATA-less promoters [21]. Since β_1_-INR shows significant sequence similarity with the TdT-INR [13] (see Figure 9A), we tested whether TAF1 and TAF2, perhaps as a cofactor for PHF1b, would influence promoter activity that is dependent on the β_1_-INR.

The transcriptional start site from β_1_-INR was analyzed in the context of a synthetic GAL4 upstream activating sequence (UAS) (Figure 9B). Promoter activity of this construct (p5XG-β_1_-INR-Luc construct) was significantly reduced in COS-7 cells when compared to a construct that contained the adenovirus E1B TATA instead of β_1_-INR [43] (data not shown). This result is to be expected given the fact that the TATA-less promoters are in general weaker than TATA-containing promoters [12] and β_1_-INR is derived from a neural specific gene.

In order to determine whether transcription initiated from the synthetic promoter through the β_1_-INR, total RNA was prepared from COS-7 cells that had been co-transfected with the expression construct for the GAL4-VP16 activator and p5XG- β_1_-INR-Luc. Primer extension analysis showed two major transcripts originating from use of the synthetic promoter (Figure 9C). The start sites we identified are different from those observed by Russek et al., [7], but both originate within the sequence of β_1_-INR. This discrepancy of start sites is most likely due to the fact that the two promoters are structurally different from each other, one being in the original human *GABRB1* promoter, studied in neurons, and the other containing only the β_1_-INR element in the context of GAL4 UAS, studied in COS-7 cells.

The p5XG-β_1_-INR-Luc construct was also used in co-expression studies with either TAF1 or TAF2 and PHF1b to study the effect of TAF co-activator properties on promoter activation. Again, the GAL4-VP16 construct was used to express the common upstream activator that recognizes the GAL4 UAS for these experiments (Figure 9B and D). Over-expression of PHF1b shows enhancement of luciferase activity with greatest effect in the presence of either TAF1 or TAF2 (Figure 9D). PHF1b over-expression potentiates TAF co-activity two to three fold. Moreover, co-expression of TAF1 and TAF2 with PHF1b increases the activity of the p5XG-β_1_-INR-Luc promoter as much as six fold compared to activation with the GAL4-VP16 activator alone.

### PHF1b represses transcription from the TK promoter at a distance

**Figure 10:**
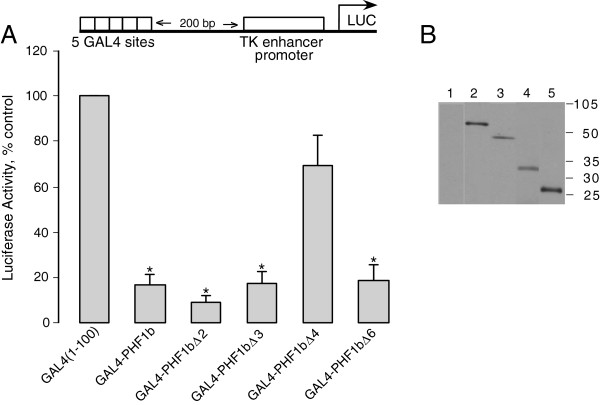
**Investigation of transcriptional repression using a panel of GAL4-PHF1b fusion proteins. A)** Full length PHF1b and truncated PHF1b versions fused to GAL4 (1–100) were recruited upstream of the TK enhancer promoter which was linked to a luciferase reporter gene (pG5-200tkLUC). GAL4 (1–100) contains the DNA binding domain that recognizes GAL4 regulatory sites. GAL4-PHF1b fusions were expressed from a CMV promoter. COS-7 cells were co-transfected with the reporter plasmid (pG5-200tkLUC) and the constructs as indicated. 48 hours after transfection, cells were harvested and assayed for luciferase activity. Results shown are mean values ± SEM and normalized to protein content within each dish as well as to vector control (pG5-200tkLUC+ GAL4 (1–100) defined as 100%). “*” indicates significantly different from vector control (p < 0.05) as determined by 95% confidence interval. **B)** GAL4-PHF1b fusion proteins are expressed in COS-7 cells as shown by Western analysis. Extracts of COS-7 cells mock transfected or expressing PHF1b constructs were analyzed by Western analysis using a GAL4 antisera. No proteins were detected in mock-transfected cells (1) and proteins were detected for cells transfected with PHF1b (2), PHF1bΔ4 (3), PHF1bΔ3 (4), PHF1bΔ6 (5). Molecular size markers are to the right.

In order to study transcriptional properties of various PHF1b domains the protein domains were individually fused to the GAL4 DNA binding domain and tested. A synthetic promoter containing the GAL4 UAS and the TK enhancer promoter was employed to test whether PHF1b could function as a repressor or activator from a distance. GAL4-PHF1b fusion constructs were co-transfected with the TK enhancer promoter construct into COS-7 cells. The GAL4-PHF1b fusions were recruited 200-bp upstream of the TK enhancer promoter at the GAL4 UAS (Figure 10) and luciferase reporter activity was measured. As seen in Figure 10, full length PHF1b fused to GAL4 functions as a strong repressor of the TK enhancer promoter from a distance. To delineate the minimum domain required for repression, we tested both amino and carboxyl terminus deletions of PHF1b (Figure 3). A fusion protein containing both PHD fingers constitutes the most potent repressor of TK promoter activity (Figure 10, PHF1b Δ2). These results also show that each PHF1b PHD finger contains a potent repressor domain (Figure 10, PHF1b Δ3 and Δ6). Interestingly, deletion of the amino terminus with PHD finger I abolishes the repressive function of PHF1b (Figure 10, PHF1b Δ4). The expression levels of these transfected GAL4-PHF1b fusions are relatively similar in COS-7 cells and confirmed with immunoprecipitation analysis by using GAL4 antibodies (Figure 10B).

### PHF1b co-immunoprecipitates with SUZ12, a PRC2 associated protein

**Figure 11:**
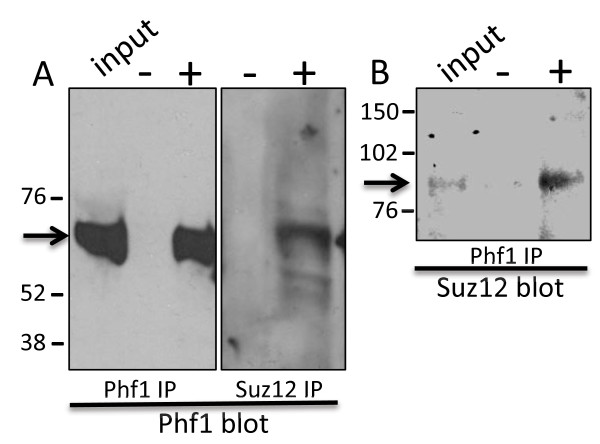
**PHF1 and SUZ12 are co-immunoprecipitated from primary neocortical neurons, as shown by Western blot.** Primary E18 cortical neurons, maintained seven days in culture, were lysed and immunoprecipitated (IP) with PHF1 (A lane 3, B lane 3), SUZ12 (A lane 5) and pre-immune (A lane 2, lane 4, B lane 2) antisera. Immune complexes were separated by sodium dodecyl sulfate polyacrylamide gel electrophoresis (SDS-PAGE) and transferred to nitrocellulose. PHF1 **(A)** or SUZ12 **(B)** proteins were identified by immunoblotting with either PHF1 **(A)** or SUZ12 **(B)** antisera. The molecular weight markers are shown to the left.

**Figure 12:**
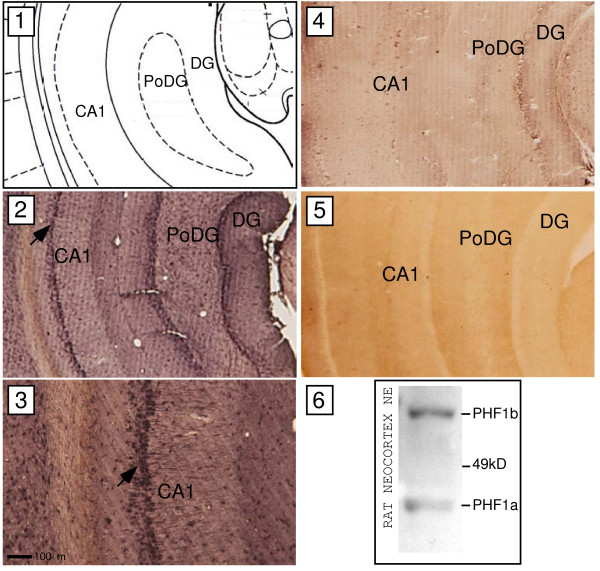
**Immunodetection of PHF1 proteins in nuclear extracts of primary rat neocortical cultures and slices of adult rat hippocampus.** Western analysis of PHF1a and PHF1b expression using nuclear extracts of rat neocortical neurons and a primary antibody raised against a PHF1 peptide present in both PHF1a and PHF1b **(panel 6)**. Relative size of PHF1 proteins is as indicated using relationship of migration pattern of putative PHF1a and b to position of marker proteins. Adult rat brains were sectioned coronally at Bregma −6.3mm **(as depicted in panel 1)** and treated with a primary antibody to PHF1 as described above. Positive immunostaining is indicated by brown-black precipitates **(panel 2 and 3)**. Regions of CA1 (field CA1 of hippocampus), DG (dentate gyrus) and PoDG (polymorph layer dentate gyrus) are indicated as references. Panel 2 displays high PHF1 immunoreactivity in the CA1 region. Arrow in panel 2 indicates area of CA1 region that was magnified (100×) in the display of panel 3. A dark scale bar at the bottom of panel 3 shows the virtual distance between two points. Hippocampal slices processed in parallel to the experimental were treated with the PHF1 antibody blocking peptide and secondary antibody **(shown in panel 4)**. A representative hippocampal slice processed in parallel treated only with the secondary antibody is displayed in **panel 5** as an additional control.

In the PRC2 complex fraction, purified from HeLa and 293F cells, PHF1a is associated with PRC2 proteins [42,44]. We now asked whether neuronal PHF1b would also be part of PRC2 by determining whether it associates with any of the key proteins of the PRC2 complex. The SUZ12 antibody was chosen for co-immunoprecipitation analysis because this protein is an integral member of the PRC2 complex and because SUZ12 affinity columns have been successfully used to isolate EZH2-EED complexes found in the HeLa cell extract [44]. In the PHF1 immunoprecipitate, upon Western blot, the larger alternatively spliced isoform b of PHF1 is detected (see Figure 1, compare PHF1a and PHF1b) (Figure 11 A, lane 1 and 3). PHF1b is also the predominant isoform detected by standard Western analysis in rat neocortical neurons (Figure 12, panel 6). In the SUZ12 immunoprecipitate, PHF1b is detected (Figure 11A, lane 5) and likewise in the PHF1 immunoprecipitate, SUZ12 is detected confirming a potential association between the proteins in neurons (Figure 11B).

### PHF1 proteins are highly expressed in the rat brain

Although PHF1a was present in the library, we did not identify PHF1a as a β_1_-INR associating factor in yeast one-hybrid assays, even though it contains significant homology to the INR binding domain of PHF1b (Figure 2). Thus, it was hypothesized that the levels of PHF1a might differ from PHF1b in neurons. To understand whether there is a significant difference in the levels of alternatively spliced versions of PHF1, a PHF1 antibody was generated against a 20 amino acid peptide sequence that is common to both PHF1 isoforms (see Methods). Western blot analysis using the PHF1 antibody was then performed with nuclear extracts derived from primary cultured E18 rat neocortical neurons and adult rat brain. Two distinct protein bands of sizes 45 kD (PHF1a) and 60 kD (PHF1b) were observed (Figure 12, panel 6) with PHF1b being the predominate splice variant.

Given the fact that many GABA_A_ receptors in the hippocampal formation are believed to contain β subunits [45], adult rat brain tissue was examined for the presence of PHF1 protein in regions where β1 subunit expression is expected to be high. Slices (Bregma –6.3mm) were stained with a PHF1 antibody as described above and in Methods. Hippocampal neurons show marked levels of PHF1 expression in the CA1 region, as well as the dentate gyrus (Figure 12, panels 2 and 3). PHF1 expression was also detected in the neocortex (data not shown).

## Discussion

A human Polycomblike protein was discovered that associates with the initiator of the core *GABRB1* promoter. The PHF1b PHD zinc finger domain (C_4_HC_3_), HLH structure and the glycine-rich motif (SFPSGQGPGGG) of this protein are sufficient for specific DNA association at β_1_-INR. Importantly, this discovery of a Polycomblike protein as a potential DNA recognition molecule for inhibitory receptor subunit expression sheds light on the important role of Polycomb group (PcG) proteins, whose mechanism for developmental regulation of transcription remains unknown [31,33,46]. In addition, our results may explain how the PcG/trxG complexes could be recruited to specific DNA sequences through protein-DNA interactions. PcG proteins were initially identified in *Drosophila* as proteins that are involved in maintaining the repression of homeotic genes necessary for anterior-posterior development [47]. PcG and trx-G are required for the maintenance of homeotic gene expression after the degradation of the gap and pair-rule proteins [47]. TrxG maintains expression of homeotic genes, whereas PcG factors maintain their repression [31].

The function of PHD fingers is not well understood. Proteins containing PHD finger motifs are believed to drive chromatin remodeling [35] by affecting protein-DNA or protein-protein interactions. Recent reports contributed to our understanding of how this domain might function in the context of some well-characterized proteins. For example, acetyltransferase activity of CBP (CREB binding protein) is dependent upon an intact PHD finger [48]. Another report [49] has shown that ING2, a PHD protein and a putative tumor suppressor protein, binds to phosphoinositides (PtdinsPs) through its PHD finger domain. The PHD finger of ING2 is a PtdinsPs nuclear receptor and is involved in nuclear responses during DNA damage. Interestingly, we find that PHF1 (variants a and b) is a nuclear protein with nuclear localization determined by the PHD finger II (Figures 4, 5).

PHF1b may represent a different class of PtdinsPs nuclear receptor proteins that are also capable of specific DNA binding. Our DNA association studies show that the PHD finger II is not sufficient for DNA association on its own, but requires another adjacent 131 amino acid region, capable of forming a HLH [36]. Apart from containing the NLS, it is not clear how the PHD finger II contributes to the overall binding of DNA. Whether the PHD finger or the HLH motif of PHF1b makes contact with the β_1_-INR-DNA remains to be determined. It is possible that the PHD finger itself may not be physically required for DNA binding but essential for modulating PHF1b’s DNA binding recognition. Alternatively, we propose that PHD fingers may provide initial DNA sequence recognition by helping interaction with nucleosomes. A similar hypothesis has been proposed by Ragvin et al. [50], who studied the function of the PHD finger in the context of a bromodomain. The authors show that both the bromodomain and PHD finger region of p300 are required for binding of acetylated nucleosomes *in vitro*. In this context, the PHD finger is thought to function as a co-recognizer of the nucleosomes or as a stabilizer of the bromodomain.

The functions of PcG and trxG proteins are mediated by overlapping Polycomb/Trithoraxgroup response elements (PRE/TRE) [31,51,52]. The mechanism behind target recognition of these sites still remains to be determined. Among the family of PcG and trxG, only three members have been shown to have specific DNA binding functions. PcG member PHO (a YY1 homolog) and two trxG proteins named GAGA and Zeste bind specific DNA sequences [53-57]. Our results suggest that PHF1b is another specific DNA binding protein of the PCL family that may function in a novel manner to recruit the PcG and trxG complexes to an INR sequence for effective control over pre-initiation complex formation at the core promoter region.

Results of PHF1b overexpression also show that activation of the human *GABRB1* promoter or a synthetic promoter containing a single β_1_-INR can be positively regulated by PHF1b (Figures 6 and 9D). This is a surprising result given that PcG proteins are usually associated with transcriptional repression. It is intriguing that PHF1b can function as a positive and negative modulator of transcription in a manner similar to the function of the YY1 protein [23,58]. The positive or negative nature of YY1 regulation is also thought to be context dependent and achieved through the interactions with specific modulatory factors [58]. PHF1a/PHF1b and YY1 protein both share zinc finger domains required for DNA binding [26]. Interestingly, the β_1_-INR is also similar to the core portion of the AAV P5+1 INR [26] promoter that is bound by YY1 (see Figure 9A). It remains to be determined whether these two initiator recognition proteins may recognize one another’s binding sites to recruit different PcG complexes. The fact that YY1 has been implicated in gene regulation of neurons [59] and that *Gabrb1* is expressed early on in the germinal matrix of the embryonic rat nervous system [60] and in the adult rat brain [45], suggests that there may be a relationship between PHF1b and YY1 regulated transcription.

Both *Drosophila* PCL and PcG protein YY1 [61] interact with the mammalian members of RPD3 family of HDACs [62,63], suggesting an involvement in chromatin remodeling. Apart from being an INR binding protein, YY1 is also expressed in the *Xenopus* anterior neural tube during tailbud stage in embryos. Inhibition of *Xenopus* YY1 function resulted in embryos with antero-posterior axial patterning defects similar to over expression of *Xenopus*PcG genes *XPCL1/2, Xbmi1* and *XEZ*[64-67]. Results of our GAL4-PHF1b fusion protein studies show that PHF1b is a strong repressor when recruited at a 200-bp distance from the TK enhancer promoter (Figure 10A). Repression by the PHF1b fusion protein is similar to that reported for the GAL4-YY1 fusion protein [23].

PHF1b-mediated repression is conferred by two PHD fingers that are also capable of repressing individually when they are fused to the GAL4 DNA binding domain (Figure 10; PHF1 Δ3 and Δ6). It has been shown that two PHD fingers of *Drosophila* Polycomblike protein are the target sites for RPD3 (histone deacetylase) interaction [62], which is consistent with our results where the repressive function of PHF1b is lost after deletion of the amino terminus of PHF1b containing the PHD finger I domain (Figure 10A; PHF1 Δ4).

The alternatively spliced version of PHF1, PHF1a, has been found to be associated with Enhancer of Zeste, EZH2 [42,44,68] to catalyze H3K27 trimethylation, which is essential for the maintenance of the repressive chromatin status of the HoxA gene [42]. The authors also found that the GAL4-PHF1b fusion protein is a strong repressor when it is recruited upstream of a TK promoter reporter gene. Unlike PHF1b, PHF1a has not been shown to bind any particular DNA sequence. Our results suggest, however, that neuronal PHF1b through its recognition of the β_1_-INR may play an active role in stabilizing gene transcription rather than repression, consistent with a recent prediction for PHF1b interaction with the ATP-dependent chromodomain helicase DNA binding protein (CHD4) [69,70]. CHD4, when it is outside of the NuRD repressor complex, can function as an activator of transcription in association with p300 histone acetyltransferase [71].

Our results suggest that this may also be the case for PHF1b which immunoprecipitates with SUZ12, a key component of the repressive PRC2 complex (see Figure 11). In our studies, loss of PHF1b from *Gabrb1-p* (as measured by ChIP) is associated with a decrease in *Gabrb1* mRNA levels [34] and a decrease in monomethylated H3K27, without a subsequent increase in trimethylated H3K27 (Figure 8B). This finding suggests that either the monomethylated form may be uniquely associated with PHF1b binding to initiators or that there is an increase in di- and trimethylation that we have not yet detected with ChIP analysis. Recent studies of PHF1, and several other related genes, have revealed that PcG gene products can also be found associated with histone H3 trimethylated at lysine 36 (H3K36me3), a chromatin mark linked to transcriptionally active genes. These results suggest that the PCL family of proteins may facilitate recruitment of PcG proteins to previously active genes, leading to de novo gene silencing [72]. We are currently pursuing these studies in the laboratory to gain a better understanding of PHF1b gene regulation in the nervous system and its potential generalizability to the regulation of other gene products critical for brain development and disease.

From our studies, and taken together with the function of YY1 described above, we propose that binding of unique PcG factors such as PHF1b to the INR may be a key element to dynamically attract the chromatin remodeling machinery to the initiation site of a gene. It is here where stabilization of the pre-initiation complex is so critical for modulating rates of transcription. Unlike cells in many other regions of the body, in neurons small changes in the expression of membrane receptor proteins can have far-reaching effects on the activity of neural networks. Taken together with the finding that GABAergic excitation promotes differentiation of hippocampal progenitor cells [73], identification of a potential relationship between chromatin remodelers, receptor activation, and the transcription and/or repression of certain neurotransmitter receptor subunit genes opens a new area of investigation that may be extremely relevant to activity-dependent gene regulation in the nervous system.

## Methods

### Antibodies

PHF1(a and b) antibody (rabbit polyclonal) was raised against the peptide RPRLWEGQDVLARWTDGLLY by Research Genetics, Inc (Huntsville, AL, USA). Antibody against H3K27me1 (Cat No. 07–448, rabbit polyclonal) was purchased from Upstate (Millipore) (Billerica, MA, USA). H3K27me3 (Cat No. ab6002, mouse monoclonal)) and SUZ12 (Cat No. ab12073, rabbit polyclonal)) antibodies were obtained from Abcam Inc (Cambridge, MA, USA). A 1–200 to 1-500 dilution of antibodywas used for immunoprecipitation experiments. For Western analysis, a 1–1000 to 1-3000 dilution of antibody was used.

### Chemicals

3-aminotriazole and GABA were purchased from Sigma-Aldrich (St. Louis, MO, USA). Yeast and tissue culture media were obtained from Invitrogen (Grand Island, NY, USA).

### *His3* Screening

The NLY2 strain of yeast carrying two integrated reporter genes (*His3* and *LacZ*) was grown in YPDA media to make competent cells according to [74]. Forty μg of adult or neonatal human brain cDNA library (Clontech, constructed from 6X106 individual bacterial colonies) was transformed into competent yeast plated on minimal media (His-, Leu-) containing 10 mM 3-aminotriazole. Approximately 500 colonies were identified from the primary screen and tested for *β-galactosidase* gene expression on X-gal containing plates. Blue colonies were isolated for further analysis in *His3* growth screens. Plasmids that were recovered from both screens were transformed back into the original yeast strain to test plasmid linkage. A control experiment was also performed in yeast to verify that the DNA binding property of PHF1b (Genebank accession: BC008834) containing clones was specific to the β_1_-INR. A chromosomally integrated reporter (*lacZ*) gene without the β_1_-INR showed no activation from expression of isolated PHF1b (data not shown).

### β-galactosidase activity

β-galactosidase activity and X-gal plate assays were performed as described in [75]. DNA sequencing was performed at the Boston University School of Medicine Genetic Core facility. Yeast strain NLY2 (gift of Dr. N. Lehming) (MATa Δgal4, gal80, ura3-52, his3-200, leu2-3, trp1, lys2) was used to integrate two reporter-carrying plasmids in yeast chromosomes. Reporter plasmids were constructed with two separate yeast-integrating vectors that carried *Trp1* and *Ura3* genes for chromosomal integration. A fragment containing three tandem initiator sites (TCGACTGCGCAGGTCCATTCGGGAAT TACTGCGCAGGTCCATTCGGGAATTA CTGCGCAGGTCCATTCGGGAATTAC) was inserted 40 nucleotides upstream of *Gal1* and *His3* TATA boxes. To determine the DNA binding function of PHF1b, the deletion constructs were made with pACT2 based candidate plasmids A4 and B37. PFU polymerase amplified PHF1b fragments were inserted into NcoI and XhoI restriction sites of the backbone vector. CMV-PHF1b was constructed by inserting full length human PHF1b cDNA in between Nhe1 and Xho1 sites of pCI-neo Vector (Promega). *GABRB1*-luciferase was previously described in [7]. 5XGAL4-INR-LUC is a derivative of the pGL2 vector (Promega). A fragment containing a single initiator with five upstream Gal4 sites was inserted in between the SmaI and BglII sites of pGL2. GAL4-VP16 expression was driven by a SV40 promoter and is described in [76]. CMV-DsRed-Nuclear and CMV-DsRed-Monomer were obtained from Clontech for nuclear localization studies. CMV-TAFII250 was a generous gift of Dr. R. Tjian. T7-TAFII150 [77] was a generous gift from Dr. R. G. Roeder and was converted to a CMV-TAFII150 with an insertion of the CMV promoter fragment (blunted BglII-SmaI). GFP fusion plasmids were constructed by inserting GFP (NheI-SalI) in between the CMV promoter and the PHF1b derivatives depicted in the figure. GAL4(1–100)/PHF1b fusions were constructed in a similar way by inserting the GAL4(1–100) fragment into the NheI and SalI sites between the CMV promoter and PHF1b fragments. All plasmids were confirmed by sequence analysis.

### Primer extension analysis

Primer extension analysis of the p5XG-INR-LUC plasmid containing the β_1_-INR was performed according to [40]. A luciferase specific primer (5′-CCATCCTCTAGAGGATAGAATGGC GCCGGG-3′) was used for primer extension analysis. Total RNA was isolated from transfected COS cells containing the p5XG-INR-Luc plasmid and GAL4-VP16 activator plasmid for analysis.

### Chromatin IP

ChIP assays were performed as previously described [78]. Five to 10 million cells were used for each assay and were split into three aliquots for immunoprecipitation in the presence and absence of PHF antibodies (200 × dilution). Genomic DNA (gDNA) was sheared to produce fragments of 300–500 bps. Average size was verified by agarose gel electrophoresis. Immunoprecipitatedg DNAs were isolated and dissolved in 100 μL TE to be used as templates for PCR amplification of a 213-bp fragment of the *GABRB1* promoter that contains the PHF1b binding site (β_1_-INR). Primers 5′-AAGGGATTGAAATCTGTTGCCTG-3′ (β_1_-forward) and 5′-CCAAACTCTCTCGATTTTGTACT-3′ (β_1_-reverse) (rat β1: Genebankaccession: AC114826). 35S-labeled PCR products were separated on a 5% polyacrylamide gel and exposed to X-ray film (Kodak). PCR was also performed on gDNAs precipitated with rabbit IgG (Santa Cruz) as a negative control that was used for normalization. Figure 7C PCR detection was performed without radioactive isotope. Real-time PCR analysis (Figure 8) was performed using primers and probe designed with SciTools (IDT). *GABRB1-p* primers: sense (5′- TGTTTGCAAGGCACAAGGTGTC -3′), antisense (5′-TCTGCGAAGATTCAAGGAATGCAACT -3′); probe: 5′FAM- TCCATTCGGGAATTACTGCCCAGCCGCCGA -TAMRA3′. Thermocycling was done using the ABI7900HT in a final volume of 20 μL. PCR parameters were 50°C for 2 min, 95°C for 15 min, 50 cycles of 95°C for 15 s, and 60°C for 1 min. Standard curves were generated from rat gDNA (Clonetech). Data were normalized as percentage of antibody/IgG signal after adjustment to input.

### Culturing and transfection of primary rat neocortical neurons

Primary cortical and hippocampal neurons were derived from 18-day rat embryos and grown in media as described [7]. Cells were plated on 100 mm tissue culture dishes (1.33 brains per dish). The plating medium was replaced by a serum-free conditioned medium after one-hour incubation. Cultures were maintained for 7–9 days before being used for transfections or ChIP experiments.

Primary cell cultures were transfected using a modified calcium phosphate precipitation method [79]. Briefly, DNA and CaCl_2_ was mixed with HeBs (137 mM NaCl, 5 mM KCI, 0.7 mM Na_2_HPO_4_, 7 H_2_O, 7.5 mM dextrose, 21 mM HEPES, pH 7.14) and stored in the dark at room temperature for 25–30 min. Cultures were washed twice with DMEM (Invitrogen, Rockville, MD) and 250 μL of a DNA precipitate were added to each dish. CMV-PHF1b or CMV-vector DNA (10 μg) were transfected into each 100 mm dish (Nunc) with 5 μg of the *GABRB1*-luciferase promoter/reporter construct. Cultures were harvested and luciferase activity was measured [80].

### Cell culture, transient transfection of COS-7 cells, fluorescent and confocal microscopy

COS-7 cells were grown in DMEM, penicillin/streptomycin, 10% fetal bovine serum (FBS), and 2 mM glutamine. COS-7 cells were grown to confluency in T flasks and treated with trypsin/EDTA. The cells were treated with 10 ml of media and seeded at 2X105/plate. After seeding (24 h), COS-7 cells were transfected using the FUGENE transfection reagent (Roche). 1.2 μl FUGENE/1 μg of DNA was used for each transfection. The expression of all plasmids used in our transfection studies was compared by Western analysis to control for nonspecific differences in functional assays that might be due to DNA quality or size of insert. Amount of DNA used in transfection assays was also based on moles rather than μg of vector DNA. After 48 hours, cells were assayed for luciferase activity (Promega kit and Victor 1420 detection system (Wallac)) or visualized with fluorescent microscopy. Luciferase counts were normalized independently to either total protein content or CMV-βgal activity. To prepare the cells for fluorescent microscopy, the plates were incubated 15 min in fixing solution: 4% paraformaldehyde, 25 mM HEPES, 150 mM NaCl, 1 mM CaCl_2_, and 1 mM MgCl_2_, washed 3× in PBS and then incubated in a quenching solution (PBS/50 mM NH_4_) for 10 min. A PBS wash followed quenching. The cells were permeabilized with PBS/1% Triton X-100 for 5 min followed by a PBS wash. Transfected COS-7 cells were visualized using a fluorescent microscope (Zeiss Axioscope) and photographed using slide film (Kodak Elite II 400). Cells for confocal imaging were plated on glass coverslip dishes (MatTek Corp). Images of primary neurons were acquired using a Zeiss Axiovert 100M laser scanning confocal microscope with a C-Apochromat 40×/1.2 water immersion objective and an optical depth of 1 μm. An argon laser was used to detect GFP and a helium-neon laser was used to detect DsRed. The photomultiplier gain and pinhole aperture were kept constant.

### Immunoprecipitation

Cortical cells were rinsed twice in ice-cold PBS and lysed in ice-cold lysate buffer (1% (v/v) Nonidet P-40, 0.1% (w/v) sodium dodecyl sulfate (SDS), 10% (v/v) glycerol, 50 mM, Tris HCl, pH 8, 150 mM NaCl with protease inhibitors (Roche)). Cell lysates were cleared by centrifugation (20,000 g, 4°C, 10 min). The supernatants were incubated overnight with specific antibody or pre-immune serum. Then, protein A Sepharose beads were added for 2 hours. The beads were washed three times with lysate buffer and once with water. Proteins were eluted by boiling in sodium dodecyl sulfate polyacrylamide gel electrophoresis (SDS/PAGE) loading buffer and separated by SDS/PAGE before Western blotting.

### Animal care

Adult male Sprague–Dawley rats (250-300 g) were purchased from Taconic Farms (Germantown, NY, USA) and housed individually with water and food available ad libitum. A 12-h light/dark cycle was maintained and all experiments were performed during the light cycle. All protocols were consistent with the guidelines of the National Institutes of Health and were approved by the Boston University School of Medicine Institutional Animal Care and Use Committee.

### Tissue collection, sectioning and antibody staining

For immunohistochemistry, rats were euthanized with 100 mg/kg pentobarbital (i.p.). The animals were then perfused through the heart with 120 ml of 0.9% saline followed by 60 ml 2% paraformaldehyde in 0.1 M PBS, pH 7.3. The brains were removed and further fixed in 2% paraformaldehyde for 48 h at 4°C. Thirty-micron coronal serial sections were obtained using a vibratome (Energy Beam Sciences, Agawam, MA, USA) and placed in PBS until being processed by single-label immunohistochemistry. Brain sections were incubated in 4% normal rabbit serum (Jackson ImmunoResearch, West Grove, PA, USA) diluted in PBS for 45 min to prevent nonspecific binding. The sections were incubated overnight in primary rabbit antibody (PHF1) diluted 1:500 in PBS at 4°C. The incubation was followed by a 30 min wash in PBS. The sections were then incubated for 2 h in biotinylated goat anti-rabbit IgG (Vector Laboratories, Burlingame, CA, USA) diluted 1:500 in PBS. The sections were rinsed in PBS for 30 min, followed by incubation in avidin-biotin-peroxidase reagent (30 min) (ABC Elite; Vector). A final rinse in PBS preceded treatment with diaminobenzidine tetrachloride (DAB) containing H_2_0_2_ and nickel-enhancing solution for 10 min. Sections were mounted using slides and Slow Fade mounting media (Molecular Probes, Eugene OR, USA). Control sections were processed as described but without primary antibody or in combination with 5-fold excess of PHF blocking peptide for 2 hours before immunohistochemistry was performed.

### Western blot analysis

Nuclear extract made from primary rat neocortical neurons [80] was electrophoresed on a 10% SDS-PAGE gel and transferred to a Biorad nylon filter (PVDF type) for Western blot analysis. The filter was probed with PHF1 antibody (1:3000 dilution) and the analysis was performed [81]. Immunoprecipitation of GAL4 fusion proteins was visualized using a polyclonal antibody raised against the GAL4 DNA binding domain (amino acids 1–147). GAL4 antibody was a kind gift Dr. Mark Ptashne.

## Conclusions

We have demonstrated that the Polycomblike protein PHF1b binds to β1-INR to stimulate transcription, and that chronic exposure to GABA reduces PHF1 binding and H3K27 monomethylation associated with transcriptional activation. This strongly suggests that PHF1b, a protein involved in homeotic gene expression in Drosophila, may be a molecular transducer of GABAAR function and thus a component of GABA-mediated neurotransmission in the human central nervous system. We have also shown that PHF1b recognition of β1-INR is dependent on a plant homeodomain, an adjacent helix-loop-helix, and short glycine rich motif, and we propose that binding of PHF1b to β1-INR represents a critical step in chromatin remodeling that may be necessary for the modulation of certain forms of transcription. Given that the GABAAR contains recognition sites for a variety of agents used in the treatment of a range of brain disorders, we suggest that additional research into the role of PHF1b in the regulation of gene expression in neurons may potentially lead to the development of novel treatments for neurological and neuropsychiatric diseases such as epilepsy and anxiety.

## Competing interest

The authors declare that they have no competing interests.

## Authors’ contributions

SS, YH, SCM, and SB carried out the molecular biological studies. SS drafted the manuscript. SJR edited the manuscript and wrote the response to reviewers. All authors read and approved the final manuscript.

## Pre-publication history

The pre-publication history for this paper can be accessed here:

http://www.biomedcentral.com/2050-6511/14/37/prepub
